# Supplementary material for: Quantitative prediction of ensemble dynamics, shapes and contact propensities of intrinsically disordered proteins
Source: PLoS Comput Biol. 2022 Sep 9;18(9):e1010036. doi: 10.1371/journal.pcbi.1010036 (PMC9491582; doi:10.1371/journal.pcbi.1010036)
Supplement: S1 Fig — (PDF) [file pcbi.1010036.s001.pdf]

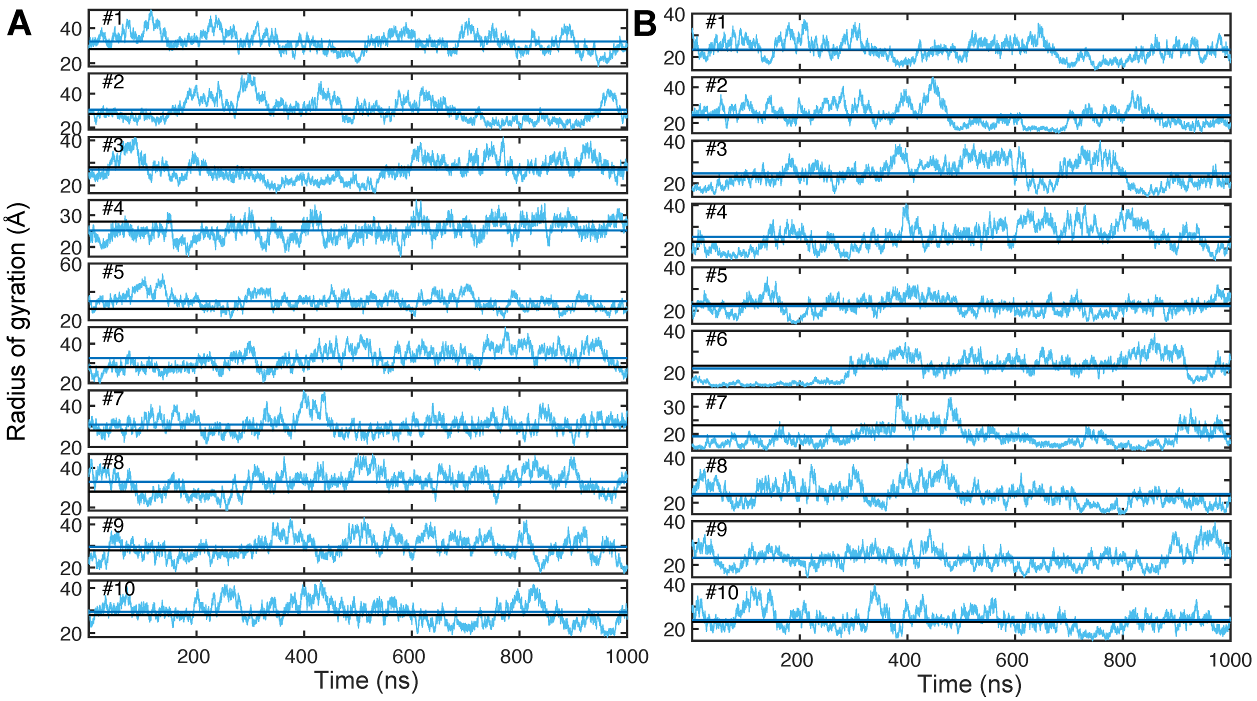

S1 Fig. Radius of gyration of the IDPs p53TAD and Pup in 10 1-μs MD trajectories each at 300 K with starting structures randomly chosen from replica-exchange simulations. The horizontal blue lines correspond to the mean R_g values for each simulation and the black lines correspond to the mean R_g determined (A) experimentally for p53TAD and (B) from the R_g-scaling power law of disordered proteins for Pup.
