# Supplementary material for: Quantitative prediction of ensemble dynamics, shapes and contact propensities of intrinsically disordered proteins
Source: PLoS Comput Biol. 2022 Sep 9;18(9):e1010036. doi: 10.1371/journal.pcbi.1010036 (PMC9491582; doi:10.1371/journal.pcbi.1010036)
Supplement: S2 Fig — (PDF) [file pcbi.1010036.s002.pdf]

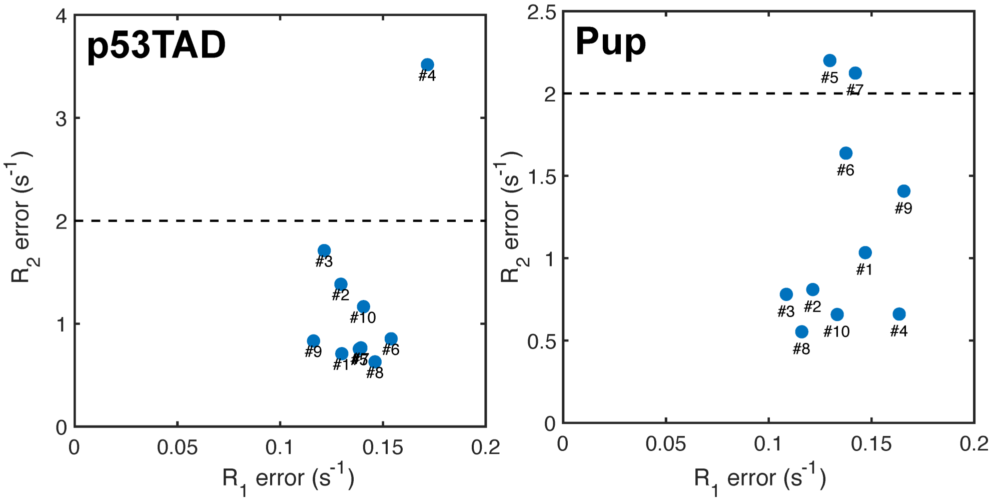

S2 Fig. Mean R_1, R_2 errors from 10 1-μs MD simulations of Pup and p53TAD in comparison with experiment. Trajectories with R_2 errors larger than 2 s^{-1} were considered as outliers, consistent with S4, S5 Tables, i.e. non-representative of the other trajectories, and excluded for the contact graph analysis in the main text (Fig 5 and 6). This concerns trajectories #5 and #7 of Pup and trajectory #4 of p53TAD.
