# Supplementary material for: Quantitative prediction of ensemble dynamics, shapes and contact propensities of intrinsically disordered proteins
Source: PLoS Comput Biol. 2022 Sep 9;18(9):e1010036. doi: 10.1371/journal.pcbi.1010036 (PMC9491582; doi:10.1371/journal.pcbi.1010036)
Supplement: S3 Fig — (PDF) [file pcbi.1010036.s003.pdf]

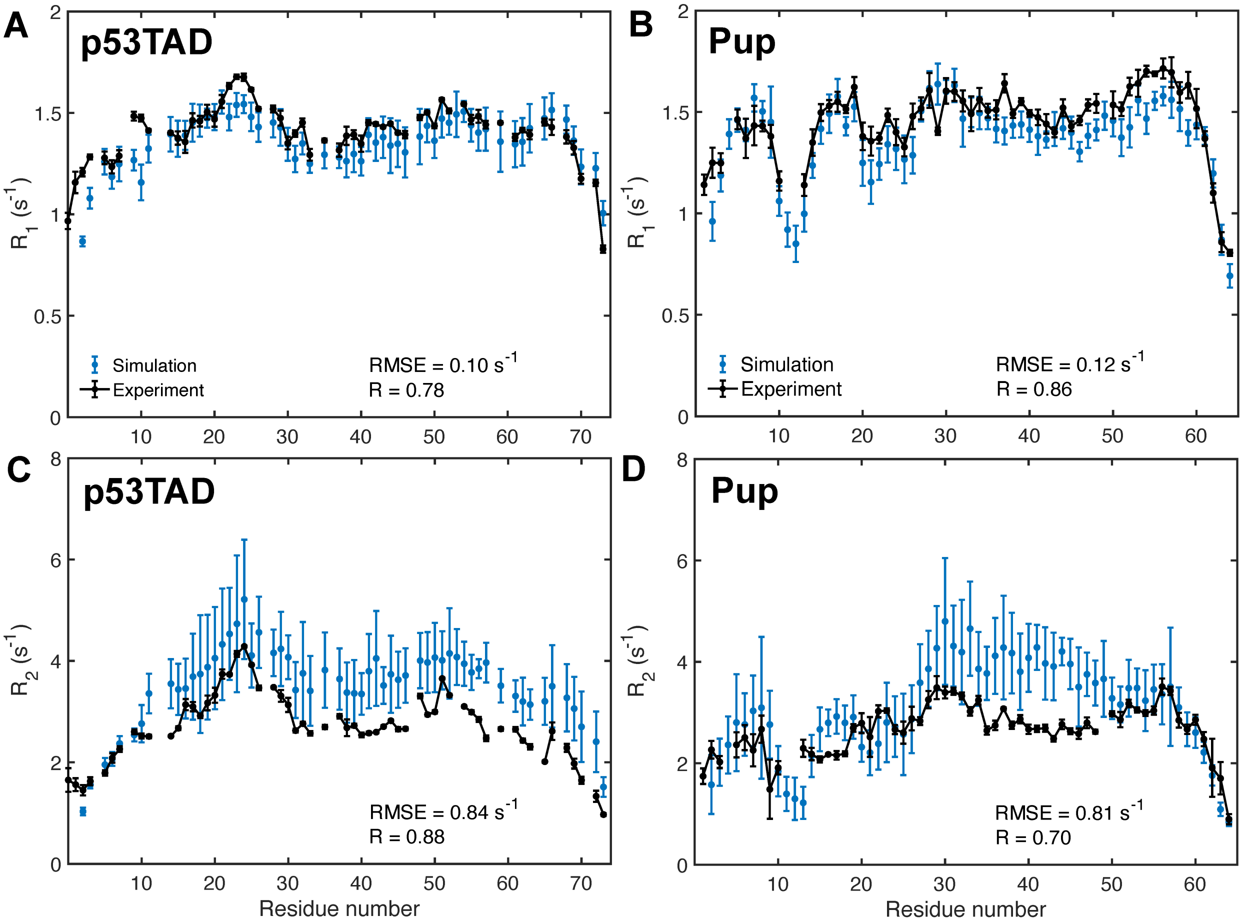

S3 Fig. Back-calculated R_1, R_2 ^{15}N backbone spin relaxation rates from microsecond MD simulations of p53TAD and Pup excluding outlier trajectories in comparison with experiment. R_1, R_2 rates calculated from average correlation functions excluding outlier trajectories (see Fig 4, S2 Fig, S4, S5 Tables) are plotted in blue with error bars showing the standard deviations among individual MD trajectories. Experimental data are plotted in black. R_1 relaxation rates determined from simulations for (A) p53TAD and (B) Pup have root-mean-square errors (RMSEs) around 0.1 s^{-1} and Pearson correlation coefficients (R) of 0.78 (p53TAD) and 0.86 (Pup). R_2 relaxation rates determined from simulations for (C) p53TAD and (D) Pup have RMSEs around 0.8 s^{-1} and correlation coefficients of 0.88 (p53TAD) and 0.70 (Pup).
