# Supplementary material for: Quantitative prediction of ensemble dynamics, shapes and contact propensities of intrinsically disordered proteins
Source: PLoS Comput Biol. 2022 Sep 9;18(9):e1010036. doi: 10.1371/journal.pcbi.1010036 (PMC9491582; doi:10.1371/journal.pcbi.1010036)
Supplement: S4 Fig — (PDF) [file pcbi.1010036.s004.pdf]

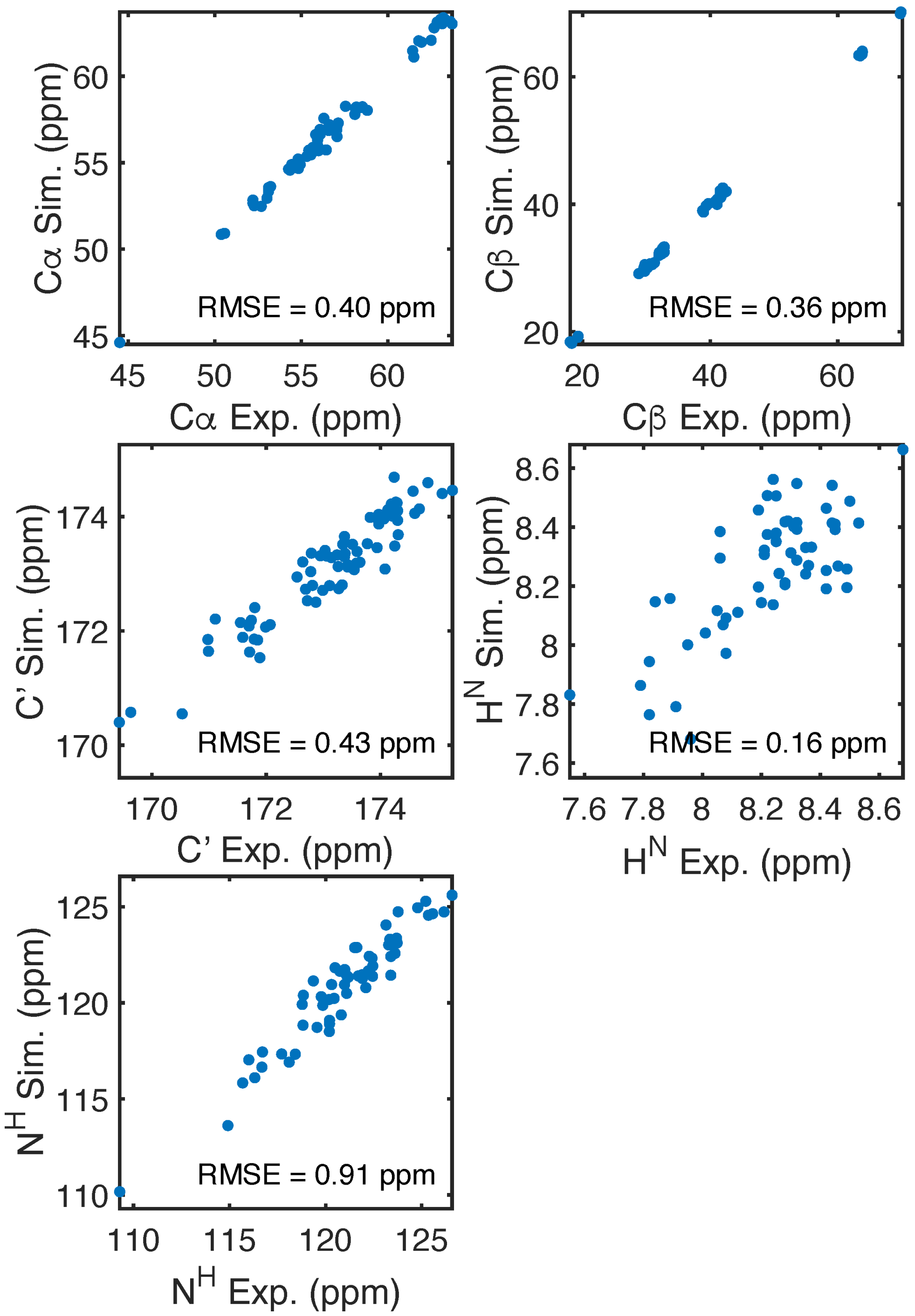

S4 Fig. Comparison of experimental and predicted chemical shifts of p53TAD. For the simulated results, Cα, Cβ, C', H^N, and N^H chemical shifts were predicted from the MD simulations (excluding outlier trajectories) using the PPM software. A reference offset correction of -3 ppm was applied to the predicted carbonyl C' chemical shifts.
