# Supplementary material for: Quantitative prediction of ensemble dynamics, shapes and contact propensities of intrinsically disordered proteins
Source: PLoS Comput Biol. 2022 Sep 9;18(9):e1010036. doi: 10.1371/journal.pcbi.1010036 (PMC9491582; doi:10.1371/journal.pcbi.1010036)
Supplement: S5 Fig — (PDF) [file pcbi.1010036.s005.pdf]

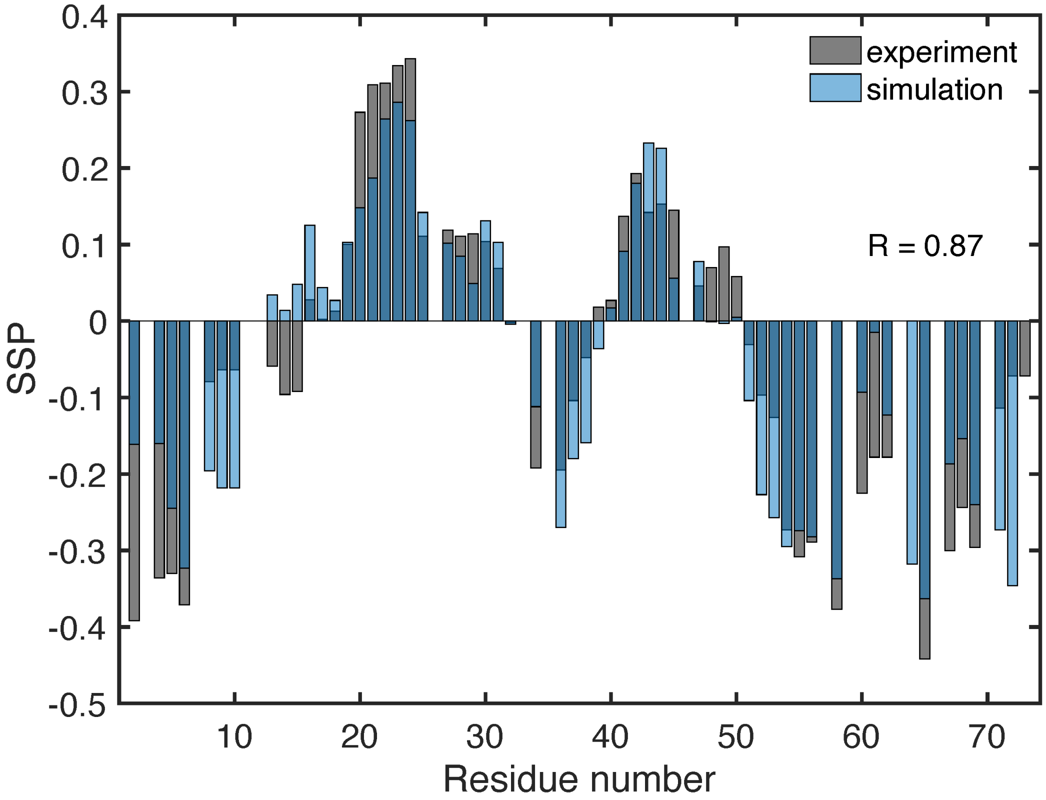

S5 Fig. Experimental and MD-derived secondary structure propensities of p53TAD. For the simulated results, secondary structure propensities were calculated from the Cα, Cβ chemical shifts predicted from the MD simulations reported here using the PPM software (see S4 Fig).
