# Supplementary material for: Quantitative prediction of ensemble dynamics, shapes and contact propensities of intrinsically disordered proteins
Source: PLoS Comput Biol. 2022 Sep 9;18(9):e1010036. doi: 10.1371/journal.pcbi.1010036 (PMC9491582; doi:10.1371/journal.pcbi.1010036)
Supplement: S6 Fig — (PDF) [file pcbi.1010036.s006.pdf]

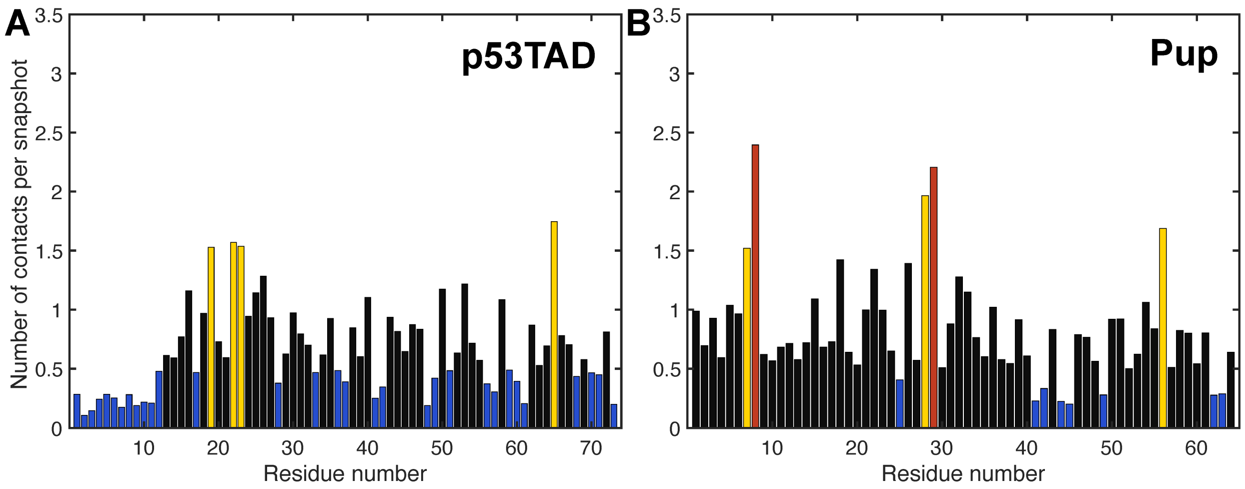

S6 Fig. Average number of contacts formed by a particular residue in p53TAD and Pup per snapshot using only side-chain atoms. Residues with average number of contacts per snapshot below 0.5 are depicted in blue, 0.5–1.5 in black, 1.5–2 in yellow, and above 2 in red. These profiles closely resemble those obtained using both backbone and side-chain atoms (Fig 5), but are systematically lower in scale.
