# Supplementary material for: Quantitative prediction of ensemble dynamics, shapes and contact propensities of intrinsically disordered proteins
Source: PLoS Comput Biol. 2022 Sep 9;18(9):e1010036. doi: 10.1371/journal.pcbi.1010036 (PMC9491582; doi:10.1371/journal.pcbi.1010036)
Supplement: S7 Fig — (PDF) [file pcbi.1010036.s007.pdf]

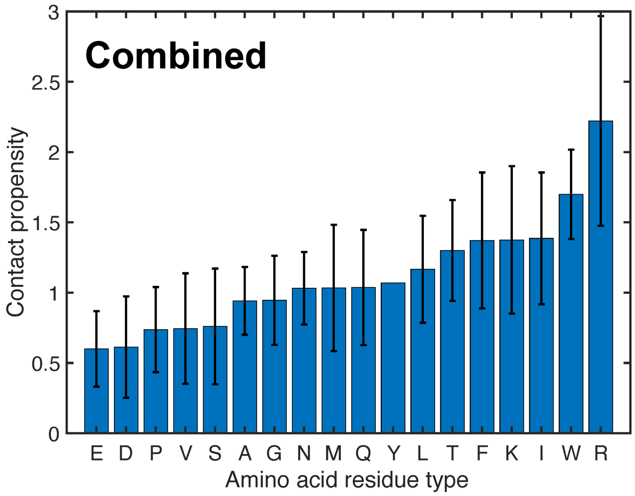

S7 Fig. Contact propensities according to amino-acid residue type for both proteins combined. The number of contacts per snapshot averaged over all residues of the same type is determined for both proteins p53TAD and Pup combined (see Fig 5 for contact propensities of individual IDPs). Error bars show the standard deviations among different residues of the same type. On average, the positively charged arginine residues are most prone to form contacts whereas the negatively charged glutamate and aspartate residues are least disposed to form contacts.
