# Supplementary material for: Quantitative prediction of ensemble dynamics, shapes and contact propensities of intrinsically disordered proteins
Source: PLoS Comput Biol. 2022 Sep 9;18(9):e1010036. doi: 10.1371/journal.pcbi.1010036 (PMC9491582; doi:10.1371/journal.pcbi.1010036)
Supplement: S1 Table — (PDF) [file pcbi.1010036.s008.pdf]

S1 Table. MD simulation details for p53TAD and Pup.

Trajectory	Simulation box size (Å^3)	Number of water molecules	Temperature (K)	Simulation time (μs)
p53TAD				
#1	117×117×117	52316	300	1
#2	120×120×120	56591	300	1
#3	103×103×103	36092	300	1
#4	97×97×97	30083	300	1
#5	128×128×128	69330	300	1
#6	103×103×103	35605	300	1
#7	120×120×120	56264	300	1
#8	125×125×125	63744	300	1
#9	82×82×82	18182	300	1
#10	97×97×97	30031	300	1
Pup				
#1	110×110×110	43275	300	1
#2	93×93×93	26647	300	1
#3	75×75×75	13484	300	1
#4	83×83×83	18785	300	1
#5	90×90×90	23621	300	1
#6	74×74×74	13485	300	1
#7	66×66×66	9263	300	1
#8	93×93×93	26647	300	1
#9	101×101×101	34280	300	1
#10	112×112×112	46011	300	1

For replica-exchange simulations, the temperature series (in Kelvin) are given below (each replica was simulated for 1 μs):
p53TAD:
298.00, 299.46, 300.93, 302.40, 303.88, 305.37, 306.86, 308.36, 309.86, 311.37, 312.88, 314.40, 315.93, 317.46, 319.00, 320.54, 322.09, 323.64, 325.20, 326.77, 328.34, 329.92, 331.51, 333.10, 334.70, 336.30, 337.91, 339.53, 341.15, 342.78, 344.42, 346.06, 347.70, 349.36, 351.02, 352.69
Pup:
298.00, 299.77, 301.54, 303.33, 305.12, 306.92, 308.73, 310.55, 312.38, 314.21, 316.05, 317.91, 319.77, 321.64, 323.52, 325.40, 327.30, 329.20, 331.11, 333.03, 334.96, 336.90, 338.85, 340.81, 342.77, 344.75, 346.73, 348.73, 350.73, 352.74, 354.76, 356.80, 358.84, 360.89, 362.95, 365.02
