# Supplementary material for: Quantitative prediction of ensemble dynamics, shapes and contact propensities of intrinsically disordered proteins
Source: PLoS Comput Biol. 2022 Sep 9;18(9):e1010036. doi: 10.1371/journal.pcbi.1010036 (PMC9491582; doi:10.1371/journal.pcbi.1010036)
Supplement: S2 Table — (PDF) [file pcbi.1010036.s009.pdf]

S2 Table. Most frequent pairwise residue contacts in p53TAD from MD simulations.

Frequency	Residue 1	Residue 2	Separation[a]
0.45	22	19	3
0.42	50	47	3
0.41	62	59	3
0.40	30	27	3
0.39	43	40	3
0.38	24	21	3
0.37	25	22	3
0.36	19	16	3
0.31	62	58	4
0.29	53	50	3
0.28	65	58	7
0.28	38	35	3
0.28	30	26	4
0.28	50	46	4
0.27	31	28	3
0.27	63	58	5
0.26	63	60	3
0.26	18	15	3
0.26	29	26	3
0.25	23	20	3
0.25	22	18	4
0.25	26	23	3
0.24	65	62	3
0.23	64	58	6
0.22	16	13	3
0.21	31	27	4
0.21	23	19	4

0.21	65	57	8
0.20	25	21	4

[a] Separation is the distance in sequence between two residues that form a contact. Contacts with separation larger than 5 residues are shaded in gray.
