# Supplementary material for: Quantitative prediction of ensemble dynamics, shapes and contact propensities of intrinsically disordered proteins
Source: PLoS Comput Biol. 2022 Sep 9;18(9):e1010036. doi: 10.1371/journal.pcbi.1010036 (PMC9491582; doi:10.1371/journal.pcbi.1010036)
Supplement: S3 Table — (PDF) [file pcbi.1010036.s010.pdf]

S3 Table. Most frequent pairwise residue contacts in Pup from MD simulations.

Frequency	Residue 1	Residue 2	Separation[a]
0.51	32	29	3
0.43	26	23	3
0.33	46	43	3
0.32	54	51	3
0.32	51	48	3
0.31	59	56	3
0.31	26	22	4
0.31	21	18	3
0.29	6	3	3
0.29	18	15	3
0.28	33	29	4
0.28	36	33	3
0.28	61	58	3
0.27	64	61	3
0.25	11	8	3
0.25	56	53	3
0.25	12	8	4
0.24	8	5	3
0.24	15	8	7
0.24	50	47	3
0.23	51	47	4
0.23	34	31	3
0.23	13	8	5
0.22	56	52	4
0.22	54	50	4
0.21	14	8	6
0.21	22	18	4
