# Supplementary material for: Quantitative prediction of ensemble dynamics, shapes and contact propensities of intrinsically disordered proteins
Source: PLoS Comput Biol. 2022 Sep 9;18(9):e1010036. doi: 10.1371/journal.pcbi.1010036 (PMC9491582; doi:10.1371/journal.pcbi.1010036)
Supplement: S4 Table — (PDF) [file pcbi.1010036.s011.pdf]

S4 Table. Chemical shifts comparisons for p53TAD

Trajectory	Cα (ppm)	Cβ (ppm)	C' (ppm)	H^N (ppm)	N^H (ppm)
PPM					
#1	0.42	0.43	0.44	0.16	0.94
#2	0.49	0.50	0.48	0.20	1.37
#3	0.49	0.37	0.41	0.19	1.11
#4	0.67	0.85	0.54	0.38	2.18
#5	0.40	0.42	0.42	0.16	0.97
#6	0.47	0.47	0.47	0.20	1.17
#7	0.48	0.47	0.44	0.20	1.30
#8	0.44	0.39	0.43	0.18	1.10
#9	0.46	0.41	0.46	0.17	1.15
#10	0.44	0.57	0.43	0.20	1.36
SHIFTX2					
#1	0.38	0.41	0.54	0.17	1.24
#2	0.42	0.53	0.57	0.19	1.39
#3	0.46	0.41	0.56	0.20	1.47
#4	0.65	0.81	0.65	0.32	2.04
#5	0.37	0.45	0.54	0.16	1.23
#6	0.41	0.44	0.57	0.19	1.43
#7	0.38	0.45	0.55	0.20	1.45
#8	0.41	0.44	0.56	0.18	1.26
#9	0.40	0.45	0.58	0.18	1.40
#10	0.40	0.55	0.55	0.19	1.42

The chemical shift prediction errors (RMSDs) using PPM for trajectory #4 (shaded row) are substantially higher than those for the other nine trajectories. For each nuclear spin type, the difference between trajectory #4 and the average of the other nine trajectories is larger than the standard deviation among the nine trajectories. These findings are not specific for chemical shift prediction by PPM as the results, e.g. using the SHIFTX2 software (bottom column), are sufficiently similar to those from PPM.
