# Supplementary material for: Quantitative prediction of ensemble dynamics, shapes and contact propensities of intrinsically disordered proteins
Source: PLoS Comput Biol. 2022 Sep 9;18(9):e1010036. doi: 10.1371/journal.pcbi.1010036 (PMC9491582; doi:10.1371/journal.pcbi.1010036)
Supplement: S5 Table — (PDF) [file pcbi.1010036.s012.pdf]

S5 Table. Radius of gyration of p53TAD and Pup

Trajectory	Initial structure (Å)	Ensemble average (Å)
p53TAD [a]		
#1	30.43	32.40
#2	29.90	30.48
#3	24.04	26.96
#4	25.96	25.21
#5	28.79	33.44
#6	30.69	32.61
#7	30.66	30.91
#8	27.63	32.94
#9	23.14	29.56
#10	22.93	29.41
Pup [b]		
#1	29.40	23.39
#2	19.72	24.31
#3	17.59	24.73
#4	19.64	25.46
#5	23.08	22.13
#6	24.98	21.89
#7	15.98	19.11
#8	23.32	23.84
#9	28.46	23.25
#10	29.41	24.02

[a] Although for p53TAD, the initial structure of trajectory #4 (shaded row), which was randomly selected from REMD, does not have the smallest radius of gyration (R_g), the ensemble average $\langle R_g \rangle$ is the smallest of all 10 trajectories of p53TAD. Thus, trajectory #4 has on average the most compact structure among all trajectories.

[b] The initial structure of trajectory #7 (shaded row) of Pup is exceptionally compact (R_g = 15.98 Å) and the ensemble average $\langle R_g \rangle$ is the smallest compared with all other trajectories of Pup. Based on the radius of gyration alone, trajectory #5 is comparable to the other trajectories of Pup.
